# Treatment of Sensitive Dentine

**Published:** 1860-10

**Authors:** 


### ARTICLE II.

### *Treatment of Sensitive Dentine.*

We can treat this subject no longer as one, the course of which is doubtful, as it has become universally recognized by practitioners who have any just claim to science, that arsenic in some available or modified manner, is the one best suited by its qualities as a chemical agent acting upon the vitality of any part, as well as by its suitability in form or substance. The common method adopted in its application has been generally in the form of arsenious acid mixed with acet. of morphia equal parts. In this way it acts as a rapid and destructive agent, too much so to admit of it in the mere reduction of the sensibility of inflamed or sensitive dentine. The entire destruction of the vitality of dentine is not required in order to prepare cavities for filling, but a reduction of sensibility sufficiently so to admit of the requircd manipulative force, so that when a filling is accomplished, the superficial injury may be renewed by the recuperative efforts of the tooth. Of this power there can be but little doubt entertained, evidenced in examples of greater destruction being remedied by it, as in secondary dentine, and in the fact of the frequent subsidence of pain from carious disease.

That arsenious acid can be applied in all cases successfully for sensitive dentine, is the object of this article, the only question remaining is how and in what manner, for in the majority of cases, the requirements vary, perhaps according to constitutional proclivities, to certain forms of inflammation, or to natural repulsion of this metal oxyd, as is found in its medical administration. Again it will vary as to amount of the ingredient, as also to the duration of time it is used, these latter points qualifying one another, throwing results almost entirely upon the experience, judgment and acumen of the practitioner, but of its availability in all cases of any ordinary form of cavity, we assert, the question to be settled is simply how must it be used? Cobalt is not crude arsenic, as is so commonly thought, but a metal oxyd always containing large proportions of arsenic in its native condition, and has been used for the above purpose from the effect produced.

Arseniate of potash is perhaps the most powerful form of any application for this purpose, and must be used very guardedly and very much diluted or modified. Arsenious acid and morphia almost as much so, but either can accomplish the reduction of extreme sensibility in dentine safely, by working them up in warm wax and applying in this form to the cavity for a series of days according to the effect produced and desired. Where extreme sensitiveness is found, it is best to use one-fourth part of the crystals powdered in bulk, to four of white wax, and to carefully examine the cavity day by day, preparing it more or less at each examination, and when sufficiently so if creasote is allowed to remain in one day, the next filling can be safely resorted to without pain.

Crude cobalt is perhaps the best to use in this way. The little portions of stony matter must be removed from the cobalt and reduced to a powder, then softened wax is made to involve as much of this powder as it will hold in a plastic condition, and applied in this form to the tooth, the cavity previously dried, when it will be found to adhere forcibly, and if not too strong, without pain, which will generally follow a too powerful application, creasote being required to subdue the too rapid irritation produced ; the object being to induce, as it were, an insensible action upon the parts subduing the pain arising from foreign contact, but not to destroy its vitality, and if this application is made skillfully it will accomplish it perfectly, often proved by the return of the former sensibility when the application has been withdrawn for a few days, and the tooth neglected.

It must be pursued with a systematic regularity, or else trouble may ensue from mismanagement, and not from the objectionable nature of the material; for instance, if the mixture has been allowed to remain too long and a filling inserted immediately upon its removal, the tooth will become discolored from its bad treatment. A pain of a violent character may ensue from a too strong application. The vitality of the tooth itself may be destroyed. These are consequences of unskillful use of the material, as a patient in pneumonia may be most effectually relieved of all suffering, or he may be destroyed by injudicious or excessive depletion. Thus the true use of arsenious acid is in all cases safely applicable, if its use is carefully and skillfully managed.

No doubt difficulties may arise to those unaccustomed to its management, but for years it has proved to be an universally available agent in avoiding excessive pain, successful operations coming daily under notice, experience fully warranting its advocacy.

The want of time is the only necessity for pain in filling teeth. Patients often require much accomplished in a given time, which necessitates immediate operation, therefore unavoidable pain, but with the privilege of time, there can be no justification of this. Cheap operators, itinerants, money jobbers, and speech makers, may object to this position, but facts and experience establish it beyond controversy. Let every skillful manipulator try it carefully and he will find results establish the truth, that all supersensitive dentine can be safely and surely reduced by this modified use of arsenious acid.

